# Family influence on the consumption of sugary drinks by children under two years old

**DOI:** 10.1590/S1518-8787.2017051000038

**Published:** 2017-06-01

**Authors:** Patricia Constante Jaime, Rogério Ruscitto do Prado, Deborah Carvalho Malta

**Affiliations:** I Departamento de Nutrição. Faculdade de Saúde Pública. Universidade de São Paulo. São Paulo, SP, Brasil; II Departamento de Vigilância de Doenças e Agravos Não Transmissíveis e Promoção da Saúde. Secretaria de Vigilância em Saúde. Ministério da Saúde. Brasília, DF, Brasil; III Departamento de Enfermagem Materno Infantil e Saúde Pública. Escola de Enfermagem. Universidade Federal de Minas Gerais. Belo Horizonte, MG, Brasil

**Keywords:** Infant, Beverages, Dietary Sucrose, Food Consumption, Diet Surveys, Health Surveys, Lactente, Bebidas, Sacarose na Dieta, Consumo de Alimentos, Inquéritos sobre Dietas, Inquéritos Epidemiológicos

## Abstract

**OBJECTIVE:**

To evaluate the influence of family habits and household characteristics on the consumption of sugary drinks by Brazilian children under two years old.

**METHODS:**

This was a cross-sectional study that used secondary data generated by the National Health Survey (PNS) in 2013. We studied 4,839 pairs of children under two years old and adults living in the same house. We estimated the prevalence of the indicator of sugary drinks consumption for the total sample of children and according to family and household variables. We applied multiple logistic regression analysis to evaluate the influence of family habits and household characteristics on the consumption of sugary drinks by the children.

**RESULTS:**

The consumption of sugary drinks was identified in 32% of the studied children (95%CI 30.6-33.3) and was independently associated with the following family and household characteristics: regular consumption of sugary drinks by the adult living in the house (OR = 1.78; 95%CI 1.51-2.10), watching TV for more than three hours per day (OR = 1.22; 95%CI 1.03-1.45), older age (OR = 3.10; 95%CI 1.54-6.26), greater education level (OR = 0.70; 95%CI 0.53-0.91), house located in the Northeast region (OR = 0.65; 95%CI 1.54-6.26), and number of family members (OR = 1.05; 95%CI 1.00-1.09).

**CONCLUSIONS:**

Our findings indicate the high prevalence of sugary drinks consumption by Brazilian children under two years old and show that sociodemographic characteristics and family habits affect this feeding practice not recommended in childhood.

## RESUMO

**OBJETIVO:**

Avaliar a influência de hábitos familiares e características do domicílio sobre o consumo de bebidas açucaradas em crianças brasileiras menores de dois anos.

**MÉTODOS:**

Estudo transversal que utilizou dados secundários gerados pela Pesquisa Nacional de Saúde (PNS), em 2013. Foram estudados 4.839 pares de crianças menores de dois anos e adultos residentes no mesmo domicílio. Foram estimadas as prevalências do indicador consumo de bebidas açucaradas para a amostra total de crianças e segundo categorias de variáveis familiares e do domicílio. Aplicou-se análise de regressão logística múltipla para avaliar a influência de hábitos familiares e características do domicílio sobre o consumo de bebidas açucaradas pelas crianças.

**RESULTADOS:**

O consumo de bebidas açucaradas foi identificado em 32% das crianças estudadas (IC95% 30,6–33,3) e esteve independentemente associado com as seguintes caraterísticas familiares e domiciliares: consumo regular de bebidas açucaradas pelo adulto residente no domicílio (OR = 1,78; IC95% 1,51–2,10), hábito de assistir TV por mais de 3 horas diárias (OR = 1,22; IC95% 1,03–1,45), maior idade (OR = 3,10; IC95% 1,54–6,26), maior escolaridade (OR = 0,70; IC95% 0,53–0,91), domicílio localizado na região Nordeste (OR = 0,65; IC95% 1,54–6,26) e número de componentes da família (OR = 1,05; IC95% 1,00–1,09).

**CONCLUSÕES:**

Os achados apontam a alta prevalência de consumo de bebidas açucaradas em crianças brasileiras menores de dois anos e que características sociodemográficas e hábitos familiares influenciam essa prática alimentar não recomendada na infância.

## INTRODUCTION

Childhood obesity is recognized as an emerging public health problem by the increase in its prevalence, its negative impact on the physical and psychological health of children^1^, and the associated risk of developing chronic noncommunicable diseases in other stages of life^2^. Efforts to prevent it pass through actions to encourage breastfeeding, promote healthy behaviors in childhood feeding and physical activity and in family food security, and regulate the food industry, aiming to protect children from incentives to inappropriate and exaggerated consumption of low nutritional quality food^3^.

In Brazil, in 2014, the National Council for the Rights of Children and Adolescents (Conanda), composed of civil society entities and ministries of the Federal Government, approved a resolution prohibiting advertising directed at children, including food advertising. For Conanda, children’s advertising hurts what is foreseen in the Federal Constitution, in the Child and Adolescent Statute, and in the Consumer Protection Code, by taking advantage of the natural vulnerability of children and their inability to analyze the interests linked to the advertising piece^a^.

In this sense, greater attention has been given to the negative impact of the consumption of sugary drinks, such as soft drinks and artificial juices and refreshments, on the diet quality^4^. Sugary drinks have low nutritional quality (restricted to the provision of sugars, such as sucrose and fructose), do not provide the same sense of satiety that solid foods do^5^, have intense advertising in the various types of media^4^, are associated with excess calories ingested in the diet, and also to overweight and obesity in children and adolescents^6^. According to the *Guia Alimentar para a População Brasileira* (Dietary Guidelines for the Brazilian Population), soft drinks and other industrialized sugary drinks are ultra-processed products and, therefore, should be avoided for an adequate and healthy feeding^7^.

The consumption of sugary drinks has an increase tendency in many parts of the world, including Brazil^4^. In the sample of 34,003 individuals with 10 years old or older who responded to the National Food Survey (INA), a module of the National Household Budget Survey of 2008-2009, the rate of consumption of juices and refreshments (39.8%) and soft drinks (23%) occupied, respectively, the sixth and ninth positions among the 20 foods with higher consumption prevalence in the Country^8^. The *Pesquisa Nacional de Demografia e Saúde* (PNDS – National Demographic and Health Survey), held in 2006, investigated indicators of breastfeeding and feeding of children under five years old, and found that daily consumption of soft drinks presented prevalence equal to 22.1% (95%CI 19.5–24.8)^9^. In the same direction, the II Survey of Breastfeeding Prevalence in Brazilian State Capitals and Federal District identified that soft drinks were early introduced in the feeding of babies from 9 to 12 months (11.6%; 95%CI 10.5–12.8)^10^.

The consumption of sugary drinks in early childhood is worrisome, especially because it is a period of formation of eating habits that can affect future eating patterns^3,11^. As mentioned by Savage et al.^12^, in these first years of life children learn what, how, when, and how much to eat, according to the social and alimentary environment to which they belong and based on the cultural transmission of beliefs, attitudes, and practices around food. Families play an important role in the development of children’s eating habits. The first social influence on human feeding comes from the familiar nucleus^13^. Parents’ food practices and behaviors, as well as socioeconomic factors of the family, have been linked to the consumption of sugary drinks between children^14-16^.

The better understanding of the factors associated with the consumption of sugary drinks by children can subsidize the development of interventions for promoting healthy nutrition and preventing childhood obesity. The National Health Survey (PNS), held in 2013, allows analyzing updated data related to infant feeding, because it included Brazilian children under two years in its population sample^17^.

This study aimed to evaluate the influence of family habits and household characteristics on the consumption of sugary drinks by Brazilian children under two years old.

## METHODS

### Study Design and Population

In this cross-sectional study, we used secondary data generated by PNS, carried out in partnership between the Brazilian Ministry of Health, Oswaldo Cruz Foundation, and the Brazilian Institute of Geography and Statistics (IBGE), in 2013. PNS is a population-based household survey that belongs to the Integrated System of Household Surveys (SIPD) of IBGE. From the master sample of that system, primary sampling units were defined, having as reference information from the Demographic Census of 2010. Thus, the sample of PNS represents Brazil, its macro-regions, urban and rural population, and capitals. Data collection took place between August 2013 and February 2014.

PNS used conglomerate sampling in three stages, with stratification of the primary sampling units. The census tracts or set of tracts were the primary units; the households were the second-stage units; and the residents aged 18 years or older, third-stage units. Records of interviews were obtained in 64,348 households. Within each selected household, a resident aged 18 years or older was selected, by simple random sampling, from the list built at the moment of the interview. 60,202 individuals were interviewed, which resulted in a non-response of 8.1%. The interviews were made using handheld computers and conducted by collection agents, under the responsibility of IBGE.

Children under two years old were identified in the second stage, when a fixed number of permanent private homes was selected in each primary sampling unit by simple random sampling, by the National Registry of Addresses for Statistical Purposes. All children of this age living in the selected households were investigated.

Sampling weights were defined for the primary sampling units, for the households and all their residents. Methodological details of PNS are described in a previous study^17^.

In this study, we analyzed 4,839 pairs of children under two years old and adults interviewed in the same house. PNS was approved by the National Human Subject Research Ethics Committee of the Brazilian Ministry of Health (Opinion 328,159/2013).

### Dependent Variable

We estimated the prevalence of sugary drinks consumption by children under two years old. The module L of the PNS questionnaire refers to the health of children in this age group, and the information is given by the mother or guardian for the child in the house. This module presented, in addition to questions about the use of health services and preventive care, a food frequency questionnaire regarding consumption in the previous 24 hours. We estimated the consumption of sugary drinks from two questions in module L – Child under two years old, of the PNS questionnaire: “Can you tell me if [name] has ingested artificial juices from yesterday morning until this morning?” or “Can you tell me if [name] has ingested soft drinks since yesterday morning until this morning?” We identified consumption of sugary drinks by the child when there was positive response in at least one of the two reference questions in the questionnaire.

### Independent Variables

We analyzed data on sociodemographic characteristics and eating behavior, practice of physical activity, weight, height, smoking, among others, of the adult (aged 18 years or older) living in the child’s house, who was taken as proxy of the behavior of other adults in the house and of the child’s family. The variables studied were: regular consumption of sugary drinks (soft drink or artificial juice consumption five or more days/week); smoking (current use of tobacco smoking); sufficient physical activity (practice of 150 minutes/week of physical activity in leisure, at work, or in displacement); habit of watching television (for three or more hours/day); overweight (body mass index [BMI] greater than or equal to 25 kg/m^2^, estimated from reported weight and height data); sex (male; female); age (18-29; 30-59; 60-64; 65 years or older); and education level (no education/some elementary or middle school; elementary or middle school/some high school; high school/some higher education; and higher education degree).

We also analyzed the following household characteristics: place of residence (urban; rural); region (Midwest; South; Southeast; Northeast; North); and number of household members.

### Data Analysis

We estimated the prevalence of the indicator sugary drinks consumption for children under two years old, presented in percentage and respective 95% confidence intervals (95%CI), for the total population and according to categories of the variables of family habits and household characteristics. Subsequently, we carried out bivariate analysis, calculating the crude odds ratio (OR), employing the Pearson’s Chi-square test with a 0.05 significance level. Lastly, we applied multiple logistic regression analysis to evaluate the influence of family habits and household characteristics on the consumption of sugary drinks by the children. We inserted in the multiple model the independent variables that were associated with the outcome at the level of p < 0.20, estimating the adjusted OR and 95%CI. We carried out data analyses using the Stata software, version 11.0. We used the survey command to analyze data from complex sample.

## RESULTS

From the information obtained in PNS, we observed that the consumption of soft drinks the day before was reported for about one out of three children studied (32.0%; 95%CI 30.6–33.3). The adults investigated were mostly women (61.9%), aged between 30 and 59 years (49.7%). About a quarter of adults that made up the pair of interest – child under two years old and adult living in the same house – reported regular consumption of sugary drinks (27%). Urban households were more frequent than those located in rural area (79.1% versus 20.9%), as Table 1 shows.

**Table 1:** Characterization of the studied population and households. National Health Survey, Brazil, 2013.

Variable	n	%
**Children under 2 years old (n = 4,839)**		
Consumption of sugary drinks (children < 2 years)		
Yes	1,569	32.0
No	3,270	68.0
**Adults (n = 4,839)**		
Sex		
Male	1,843	38.1
Female	2,996	61.9
Age (years)		
18-29	2,274	47.0
30-59	2,404	49.7
60-64	60	1.2
≥ 65	101	2.1
Education level		
Illiterate/some elementary or middle school	1,610	33.3
Elementary or middle school/some high school	1,008	20.8
High school/some higher education	1,674	34.6
Higher education degree	547	11.3
Regular consumption of sugary drinks (> 5 days/week)		
Yes	1,308	27.0
No	3,531	73.0
Smoking		
No	4,149	85.7
Yes	690	14.3
Physical activity (> 150 min/week)		
Active	2,387	49.3
Inactive	2,452	50.7
Watching TV (> 3 hours/day)		
No	3,246	67.1
Yes	1,593	32.9
Body mass index (kg/m^2^)		
< 25	1,567	49.1
≥ 25	1,626	50.9
**Household**		
Place of residence		
Urban	3,812	79.1
Rural	1,006	20.9
Region		
North	1,417	29.3
Northeast	1,448	29.9
Southeast	867	17.9
South	507	10.5
Midwest	600	12.4

This study’s hypothesis was that family habits and household characteristics would be associated with the consumption of sugary drinks by Brazilian children under two years old. As Table 2 shows, we observed higher prevalence of consumption in children whose respective adult regularly consumed sugary drinks (41.8%; 95%CI 39.2–44.5), when compared to those pairs whose adults did not consume them (28.0%; 95%CI 25.4–30.8%). We observed minor differences, according to categories of the other independent variables.

**Table 2:** Prevalence of sugary drinks consumption by children under two years old, according to family habits and household characteristics. National Health Survey, Brazil, 2013.

Variable	%	95%CI
Consumption of sugary drinks (children < 2 years)	32.0	30.6–33.3
Family habits*		
Regular consumption of sugary drinks (> 5 days/week)		
No	28.0	25.4–30.8
Yes	41.8	39.2–44.5
Smoking		
No	31.4	27.9–35.1
Yes	35.0	31.6–38.6
Physical activity (> 150 min/week)		
Active	33.2	30.5–36.0
Inactive	30.6	28.8–32.5
Watching TV (> 3 hours/day)		
No	30.7	28.0–33.6
Yes	34.7	32.3–37.2
Body mass index (kg/m^2^)		
< 25	29.7	26.7–32.9
≥ 25	32.8	30.6–35.1
Sex		
Male	31.4	28.8–34.1
Female	32.4	30.6–34.2
Age (years)		
18-29	32.1	20.0–47.1
30-59	31.8	19.8–46.7
60-64	32.6	17.2–52.9
≥ 65	34.0	19.2–52.7
Education level		
No education/some elementary or middle school	33.3	28.6–38.4
Elementary or middle school/some high school	35.3	30.1–40.8
High School/some higher education	30.4	26.0–35.3
Higher education degree	26.7	23.1–30.7
**Household variables**		
Place of Residence		
Urban	30.5	27.1–34.2
Rural	32.2	30.7–33.6
Region		
North	32.7	26.9–39.0
Northeast	24.3	20.1–29.2
Southeast	34.7	29.6–40.2
South	38.7	32.6–45.2
Midwest	34.7	30.1–39.7

* Regarding the adult living in the same house of the child and respondent in PNS-2013.

The results of bivariate analysis (Table 3) confirmed that the regular consumption of sugary drinks by the adult was associated with the consumption of the child (OR = 1.85; p < 0.001). Another family behavior that was associated with the children’s consumption was the daily habit of watching TV for three hours or more (OR = 1.20; p = 0.008). On the other hand, children whose adult had higher education degree had lower chance of consuming sugary drinks (OR = 0.73; p = 0.006), as well as those living in the Northeast region (OR = 0.66; p < 0.001). We highlight that we did not observed significant difference in the prevalence of the dependent variable, according to urban or rural location of the house.

**Table 3:** Association between sugary drinks consumption by children under two years old and family habits and household characteristics. National Health Survey, Brazil, 2013.

Variable	OR _not adjusted_	95%CI	OR _adjusted_*	95%CI
Regular consumption of sugary drinks (> 5 days/week)				
No	1.00		1.00	
Yes	1.85	1.62–2.11	1.78	1.51–2.1
Smoking				
No	1.00			
Yes	1.18	1.00–1.39		
Physical activity (> 150 min/week)				
Active	1.00			
Inactive	0.89	0.79–1.01		
Watching TV (> 3 hours/day)				
No	1.00		1.00	
Yes	1.20	1.05–1.37	1.22	1.03–1.45
Body mass index (kg/m^2^)				
< 25	1.00		1.00	
≥ 25	1.16	1.00–1.34	1.12	0.96–1.31
Sex				
Male	1.00		1.00	
Female	1.05	0.93–1.19	1.03	0.88–1.21
Age (years)				
18-29	1.00		1.00	
30-59	0.99	0.87–1.12	1.11	0.94–1.32
60-64	1.03	0.58–1.82	0.56	0.20–1.62
≥ 65	1.09	0.69–1.73	3.10	1.54–6.26
Education level				
No education/some elementary or middle school	1.00		1.00	
Elementary or middle school/some high school	1.09	0.92–1.29	1.06	0.84–1.34
High school/some higher education	0.88	0.76–1.02	0.86	0.70–1.05
Higher education degree	0.73	0.59–0.91	0.70	0.53–0.91
Place of residence				
Urban	1.00			
Rural	0.93	0.78–1.10		
Region				
North	1.00		1.00	
Northeast	0.66	0.53–0.82	0.65	0.48–0.86
Southeast	1.10	0.90–1.34	1.12	0.85–1.47
South	1.30	1.03–1.65	1.30	0.96–1.76
Midwest	1.10	0.83–1.45	1.05	0.74–0.49
Number of household members	1.02	0.99–1.06	1.05	1.00–1.09

* Adjusted by variables of habits of the adult living in the house of the child (regular consumption of sugary drinks, smoking, physical activity, watching TV, body mass index, education level) and of the household (region and number of members).

The associations observed in the bivariate analysis remained significant in the logistic regression analysis, which identified two other independent associations between variables of family and household habits and children’s consumption (Table 3). The chance of children under two years consuming sugary drinks was 3.1 times higher in those with adult living in the same house aged 65 or older (OR_adj_ = 3.10; p = 0.002), when compared to those with younger references (18 to 29 years). The number of household members was also associated with the consumption of sugary drinks by children (OR_adj_ = 1.05; p = 0.041).

## DISCUSSION

The *Guia Alimentar para Crianças Menores de Dois Anos* (Dietary Guidelines for Children Under Two Years Old)^18^, published by the Brazilian Ministry of Health, recommends avoiding sugar and soft drinks, among other candy, in the early years of the child’s life. Contrary to this recommendation, the prevalence of consumption of sugary drinks, recognized as a risk factor for childhood obesity^6^, was high in children under two years studied by PNS in 2013. To our knowledge, this is the first Brazilian study that relates food consumption data of children and adults living in the same house in a population-based survey, thus being possible to explore the hypothesis of the influence of family and household characteristics on the child’s consumption.

In the set of independent variables analyzed, two family habits showed strong association with the consumption of sugary drinks by children: the regular consumption of these beverages by adults and the daily habit of watching television for more than three hours. Previous studies made with children at different stages (preschool, school, and adolescence) found greater chance of sugary drinks consumption by children whose parents consume these drinks regularly^19,20^. PNS data indicate that this behavioral influence can occur even in the initial phase of life, i.e., early childhood.

Regarding the variable related to the daily habit of watching TV for over three hours, a systematic review conducted by Paes et al.^15^ identified that eight out of 12 studies that evaluated its association with the consumption of sugary drinks by children and adolescents found positive results. Causal mechanisms have been proposed to explain the relationship between sedentary behavior, such as watching TV for many hours, and unhealthy eating pattern^21^. One of the causes may be the exposure to food advertising, since soft drinks and other sugary drinks are often objects of strong marketing^4^. PNS data show that children living with adults who watch TV for more than three hours have a higher chance of consuming sugary drinks. One can see that environmental factors related to the family habit of watching TV can affect the food consumption of different members.

Concerning sociodemographic characteristics, while the adult age was positively associated with the consumption of sugary drinks by children, education level showed an inverse association, corroborating previous studies that show that, as well as in other health outcomes, the consumption of sugary drinks by children is affected by social determinants^14,15^.

The results of the analyses by household characteristics showed that the South region presented the highest prevalence of consumption of sugary drinks by children under two years old (38.7%), while in the Northeast, where we estimated the lowest prevalence, one out of four children had reported consumption for these drinks (24.3%). These findings indicate, on the one hand, regional differences, but, on the other, unveil a frequent eating habit in the Country as a whole, by the high prevalence observed in the five macro-regions studied. We found no difference between urban and rural households, which had been identified in the study of Bortolini et al.^9^ with PNDS-2006 data, showing that children living in urban areas consume soft drinks with greater frequency than those from rural areas. With the precautions necessary because of the methodological differences of the two population surveys, we can verify a significant increase in the consumption of sugary drinks by children living in the rural area – which was 9.7% according to PNDS-2006 and passed to 32.2% in PNS-2013. This increase can be explained, in part, by the improving access to food in general by Brazilian rural households and, consequently, better food and nutritional security situation, as found by the *Pesquisa Nacional por Amostragem de Domicílio* (PNAD – National Household Sampling Survey)^22^ in 2013, despite the increased presence of some foods with worst nutritional value, such as sugary drinks.

In the global agenda of prevention and control of childhood obesity, measures aimed at reducing the consumption of sugary drinks have been considered an emergency and priority. These measures include food and nutritional education activities involving parents, families, and caregivers of children, but also actions of control of the food industry, such as the regulation of food advertising and a policy of taxation of sugary drinks^3,4^. Taxation has been implemented in an innovative way in Mexico^23^ and, more recently, in the United Kingdom^24^. The promising results of the Mexican experience show that the adoption of tax on sugary drinks resulted in reduction in the purchases of these drinks in retail^25^. In fact, evaluative studies help understanding the potential impact of interventions in price and availability of sugary drinks on the pattern of food purchase and consumption of individuals and communities and the implications for public health, increasing the level of evidence to guide policies for controlling childhood obesity. These policies should not only cover health education and individual care, but also promote improvements in the food environment to support and help healthy eating practices by individuals, families, and communities.

This study presents common limitations to food consumption surveys: possible measurement bias of the usual diet, by lack of memory of the respondent; over or underestimation of food consumption; and limited validity of the data collection instruments.

Another limitation concerns the impossibility of assessing the family influence in relation to availability, accessibility, and permissiveness for infant consumption in the households, which showed association with soft drink consumption in children from 2.5 to 7 years old^14^. The availability and repeated exposure to certain foods is a conditioner of food preferences acquired in childhood^26^. Also, the adult taken as family reference to answer the questionnaire on behaviors was selected randomly; thus, for some households, this may not be the adult directly responsible for the child. The literature is more consistent in indicating the influence of parental modeling on the eating habits and behaviors of children^14,15^. Finally, the variable weight and height was omitted in 30% of informants, which may have changed the analysis of this variable, which was not significant in bivariate analysis.

On the other hand, by including children under two in its population sample, PNS allows analyzing updated data related to infant feeding.

In conclusion, our findings expose the high prevalence of sugary drinks consumption by Brazilian children under two years old, and show that sociodemographic characteristics and family habits affect this feeding practice not recommended in childhood.

## Footnotes

a Instituto Brasileiro de Defesa do Consumidor. Conanda aprova resolução que proíbe a publicidade direcionada a crianças. São Paulo: IDEC; 2014 [cited 2016 Aug 23]. Available from: http://www.idec.org.br/em-acao/em-foco/conanda-aprova-resoluco-que-proibe-a-publicidade-direcionada-a-criancas

## References

[1] Sahoo K, Sahoo B, Choudhury AK, Sofi NY, Kumar R, Bhadoria AS (2015). Childhood obesity: causes and consequences. J Family Med Prim Care.

[2] Biro FM, Wien M (2010). Childhood obesity and adult morbidities. Am J Clin Nutr.

[3] Lobstein T, Jackson-Leach R, Moodie ML, Hall KD, Gortmaker SL, Swinburn AB (2015). Child and adolescent obesity: part of a bigger picture. Lancet.

[4] Popkin BM, Hawkes C (2016). Sweetening of the global diet, particularly beverages: patterns, trends, and policy responses. Lancet Diabetes Endocrinol.

[5] Pan A, Hu FB (2011). Effects of carbohydrates on satiety: differences between liquid and solid food. Curr Opin Clin Nutr Metab Care.

[6] Malik VS, Pan A, Willett WC, Hu FB (2013). Sugar-sweetened beverages and weight gain in children and adults: a systematic review and meta-analysis. Am J Clini Nutr.

[7] Monteiro CA, Cannon G, Moubarac JC, Martins AP, Martins CA, Garzillo J (2015). Dietary guidelines to nourish humanity and the planet in the twenty-first century: a blueprint from Brazil. Public Health Nutr.

[8] Souza AM, Pereira RA, Yokoo EM, Levy RB, Sichieri R (2013). Alimentos mais consumidos no Brasil: Inquérito Nacional de Alimentação 2008-2009. Rev Saude Publica.

[9] Bortolini GA, Gubert MB, Santos LMP (2012). Consumo alimentar entre crianças brasileiras com idade de 6 a 59 meses. Cad Saude Publica.

[10] Ministério da Saúde (BR), Secretaria de Atenção à Saúde, Departamento de Ações Programáticas e Estratégicas (2009). II Pesquisa de Prevalência de Aleitamento Materno nas Capitais Brasileiras e Distrito Federal.

[11] Park S, Lin M, Onufrak S, Li R (2015). Association of sugar-sweetened beverage intake during infancy with dental caries in 6-year-olds. Clin Nutr Res.

[12] Savage JS, Fisher JO, Birch LL (2007). Parental influence on eating behavior: conception to adolescence. J Law Med Ethics.

[13] Birch LL, Fisher JO (1998). Development of eating behaviors among children and adolescents. Pediatrics.

[14] De Coen V, Vansteelandt S, Maes L, Huybrechts I, De Bourdeaudhuij I, Vereecken C (2012). Parental socioeconomic status and soft drink consumption of the child.The mediating proportion of parenting practices. Appetite.

[15] Paes VM, Hesketh K, O’Malley C, Moore H, Summerbell C, Griffin S (2015). Determinants of sugar-sweetened beverage consumption in young children: a systematic review. Obes Rev.

[16] Pabayo R, Spence JC, Cutumisu N, Casey L, Storey K (2012). Sociodemographic, behavioural and environmental correlates of sweetened beverage consumption among pre-school children. Public Health Nutr.

[17] Souza PRB, Freitas MPS, Antonaci GA, Szwarcwald CL (2015). Desenho da amostra da Pesquisa Nacional de Saúde 2013. Epidemiol Serv Saude.

[18] Ministério da Saúde (BR), Secretaria de Atenção à Saúde, Departamento de Atenção Básica (2013). Dez passos para uma alimentação saudável: guia alimentar para crianças menores de dois anos, um guia para o profissional da saúde na atenção básica.

[19] Van Lippevelde W, Velde SJ, Verloigne M, De Bourdeaudhuij I, Bere E, Jan N (2013). Associations between home- and family-related factors and fruit juice and soft drink intake among 10- to 12-year old children. The ENERGY project. Appetite.

[20] Vereecken CA, Keukelier E, Maes L (2004). Influence of mother’s educational level on food parenting practices and food habits of young children. Appetite.

[21] Robinson TN (2001). Television viewing and childhood obesity. Pediatr Clin North Am.

[22] Instituto Brasileiro de Geografia e Estatística (2014). Pesquisa Nacional por Amostra de Domicílios: segurança alimentar.

[23] Organización Panamericana de la Salud (2015). Experiencia de México en el establecimiento de impuestos a las bebidas azucaradas como estrategia de salud pública.

[24] Briggs A (2016). Sugar tax could sweeten a market failure. Nature.

[25] Colchero MA, Popkin BM, Rivera JA, Ng SW (2016). Bevarage purchases from stores in Mexico under the excise tax on sugar sweetened beverages: observational study. BMJ.

[26] Patrick H, Nicklas TA (2005). A review of family and social determinants of children’s eating patterns and diet quality. J Am Coll Nutr.

